# A telemedicine wound care model using 4G with smart phones or smart glasses: A pilot study

**DOI:** 10.1097/MD.0000000000004198

**Published:** 2016-08-07

**Authors:** Junna Ye, Yanhai Zuo, Ting Xie, Minjie Wu, Pengwen Ni, Yutian Kang, Xiaoping Yu, Xiaofang Sun, Yao Huang, Shuliang Lu

**Affiliations:** a Institute of Burns, Ruijin Hospital; b Department of Wound Healing, Shanghai Ninth Hospital, School of Medicine,Shanghai Jiao Tong University, Shanghai, China.

**Keywords:** smart glasses, smart phones, telemedicine, wound care

## Abstract

To assess the feasibility of a wound care model using 4th-generation mobile communication technology standards (4G) with smart phones or smart glasses for wound management.

This wound care model is an interactive, real-time platform for implementing telemedicine changing wound dressings, or doing operations. It was set up in March 2015 between Jinhua in Zhejiang province and Shanghai, China, which are 328 km apart. It comprised of a video application (APP), 4G net, smart phones or smart glasses, and a central server.

This model service has been used in 30 patients with wounds on their lower extremities for 109 times in 1 month. Following a short learning curve, the service worked well and was deemed to be user-friendly. Two (6.7%) patients had wounds healed, while others still required wound dressing changes after the study finished. Both local surgeons and patients showed good acceptance of this model (100% and 83.33%, respectively).

This telemedicine model is feasible and valuable because it provides an opportunity of medical service about wound healing in remote areas where specialists are scarce.

## 1 Introduction

Telemedicine emerged in the late 1950s and has been regarded to be of great value in the provision of health care.^[1]^ There are also studies demonstrating that the application (APP) of telemedicine is particularly beneficial to wound care in isolated communities and remote regions.^[2–6]^ Smart phones installed with various video APPs are now popular and widely used around the world. With a video APP, a smart phone could capture, transfer, and store videos, as well as enabling communication online with friends anywhere in the world. Phones have progressed beyond calls to incorporate Internet browsing, and a wide variety of device-based software APPs. These make it the most common acquisition terminal to conduct telemedicine even in the most remote and resource poor settings.^[7–10]^ Meanwhile, new wearable computers such as Google Glasses (Google, Inc., Mountain View, CA) and Vuzix Glasses (Rochester, NY) are interesting technology, attracting global interest from multiple professions. They can be worn like conventional glasses and allow the capture of video from the perspective of the wearer. In addition, it provides an interface to access to the Internet and communicates with others. One of its greatest advantages when used is that it is hands-free and relies mostly on voice commands.^[11–13]^ Studies have shown its potential to impact health care delivery, medical documentation, surgical training, and patients’ safety.^[14]^

First established in the 1990s by Gottmp F, the wound healing center has been a widespread and accepted concept based on a multidisciplinary platform.^[15,16]^ As a specialty needing a strong visual element, wound healing has been demonstrated to have a clear benefit from telemedicine.^[17–20]^ In China, the first wound healing center was established in Hangzhou in 2004, and then followed by Xi’an, Lanzhou, and Shanghai. Two key features of these emerging wound healing centers are their obviously centralized distribution in big cities; however, there is a lacking of wound specialists in remote areas. Within a large city like Shanghai it can be difficult for those patients who are unable to move freely to go to these central wound healing centers due to the distance required to travel.^[21,22]^ In 2011, we designed a telemedicine system utilizing fourth-generation mobile communication technology standards (4G) optical cable and high-resolution video for diagnosis and treatment of wounds between wound healing department in Ninth Hospital and community health care centers in other parts of Shanghai in a bid to overcome this problem. Having been used more than 600 times, it worked effectively and successfully.^[23,24]^ However, as the basic infrastructure is inadequate in remote areas in China, we came up with the idea to use the Shanghai telemedicine model to help improve the wound care in these areas, the effect of which is still not clear at the moment. A new telemedicine wound care model was set up using 4G net with smart glasses or smart phones between Shanghai and Jinhua, which are 328 km apart, to conduct a cohort study.

## 2 Methods

### 2.1 Study subjects

The study protocol was approved by the medical ethics committee of both participating centers and conformed to the guidelines set up in the Declaration of Helsinki. The study included 30 patients with skin defects on their lower limbs who presented to the First People's Hospital of Wucheng District in Jinhua, Zhejiang Province, China from April to May 2015. All the subjects signed the informed consent.

### 2.2 Personnel, health care departments, and facilities involved in this telemedicine system

Specialists from the Wound Healing Department of Shanghai Ninth People's Hospital affiliated to Shanghai Jiao Tong University School of Medicine offered the service of remote consultation to the First People's Hospital of Wucheng District in Jinhua. Local surgeons from the orthopedic department of First People's Hospital of Wucheng District, Jinhua were responsible for keeping each patient's medical records and implementing the treatment recommended by remote specialists. To improve the skill of wound management, local surgeons received training of basic wound healing knowledge in advance, which took approximately 4 days. After a period of training, local surgeons were tested to ensure they met the demands of wound management under the supervision of wound healing specialists in Shanghai.

**Figure 1:**
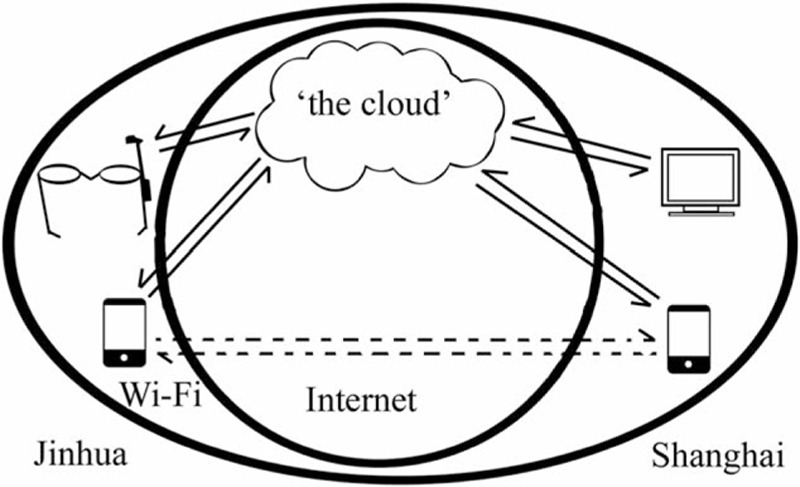
The telemedicine wound care model. A new telemedicine wound care model was set up using 4th-generation mobile communication technology standards (4G) net with smart glasses or smart phones between Shanghai and Jinhua, which are 328 km apart, to conduct a cohort study. Multiway communication between Jinhua and Shanghai can be carried out by either a 3rd party indirectly (the solid line with an arrow) or a telephone directly (the dotted line with an arrow).

The telemedicine system consisted of either a video APP based on computers and smart phones or the use of smart glasses with a central server for multiway communication. There were 3 acquisitive terminals available: computers to the specialists, smart phones to both the specialists and local surgeons, and smart glasses to local surgeons. In a 4G wireless local area network (Wi-Fi) context, the video APP allowed real-time visualization of the patients’ wound in Jinhua to specialists in Shanghai. The facilities involved were illustrated in Fig. 1.

**Figure 2:**
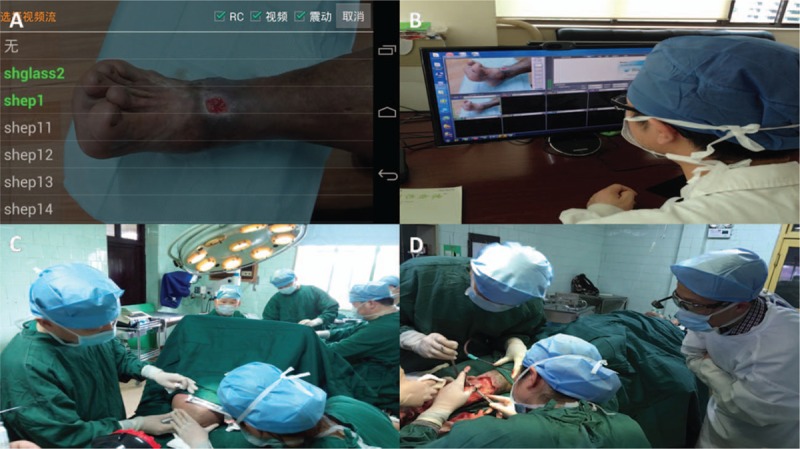
(A) A user named “shep1” in Shanghai and a user namely “shglass2” in Jinhua were both online. Then, by touching “shep1,” the real-time communication between “shep1” and “shglass2” was set up. (B) On the screen of Shanghai, the presented wound was in the smaller window in Jinhua. (C) Wound healing specialists in Shanghai went to Jinhua to help guide the operation of a 70-year-old male patient in the local hospital. (D) Shanghai experts provided real-time intraoperative consultation through the telemedicine system by smart glasses in Jinhua.

The type of smart phones used in this study was ZTE M901C (Zhongxing Telecommunication Equipment Corporation, Shenzhen, Guangdong, China), which had a 13-megapixel camera and a 6.0-inch display with a resolution of 1280 × 720 pixels. It enabled a 1920 × 1080 pixel video at the speed of 30 frames per second. The smart glasses used were Lenovo Vuzix M100 Smart Glasses (Lenovo NewBusiness Development, Beijing, China). The smart glasses consisted of a computerized central processing unit, a display screen, a high-definition camera, a microphone, a conduction transducer, and a wireless connectivity. With a 5-megapixel high-definition camera, it took videos in a resolution of 1080 pixel at the speed of 30 frames per second. The video APP was from the livecast media (Livecast Media Inc, Vancouver, BC, Canada), which enabled multiway communication including one-to-many, many-to-one, and many-to-many communication. In one case, a user named “shep1” in Shanghai and a user namely “shglass2” in Jinhua were both online and were able to use real-time communication (Fig. 2A and B).

### 2.3 Training of users’ adherence and comprehensibility

Before initiating this model, we piloted the system in healthy volunteers to evaluate feasibility of the system and to train both remote specialists and local surgeons to use the system. The average training time of local surgeons was 3 days. The acceptance of this model was asked by a YES or NO questionnaire to the local surgeons and patients.

### 2.4 The process of telemedicine system

**Figure 3:**
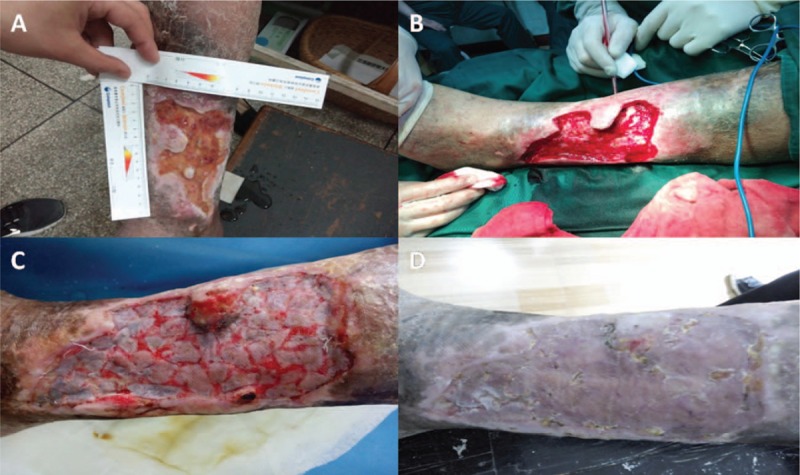
The therapeutic effect of a 72-year-old man in Jinhua with consultation from Shanghai wound healing specialists. (A) Preoperative: The wound was of an irregular shape and the size was 10 cm × 13 cm. (B) Intraoperative: Wound debridement and split skin graft was conducted by local surgeons with telemedicine. (C) One week after operation: The grafted skin was viable by 95%. (D) Three weeks after operation: The wound healed completely.

On arrival to the clinic, an experienced surgeon completed a physical examination and acquired detailed medical information of each patient. After getting the patient ready, the surgeon reported the patient's medical record to specialists. During a multiway real-time video communication, wound healing specialists in Shanghai assessed the wound accordingly and gave their expert opinions. The final management decisions were made by the specialist and the local doctor together after discussion. Afterwards, the surgeons and patients agreed another appointment for next consultation. If patients underwent surgery, wound healing specialists could provide real-time intraoperative consultation through the telemedicine system as well (Fig. 3A, B).

### 2.5 Statistical analysis

Quantitative data are presented as mean ± SD%. All statistical measurements were performed using SPSS 16.0 for windows (SPSS, Chicago, IL).

## 3 Results

In a month, the system was employed 109 times. Detailed information of patients is presented in Table 1. There were 29 male and 1 female patients with a mean age of 70.50 ± 11.08 years (ranged 40–90 years). Twelve (40%) had varicose veins. The location of the wounds were as follows: leg (n = 16, 53.33%), ankle (n = 8, 26.67%), and foot (n = 6, 20%). All patients were treated using this wound care model with the use of smart glasses of 5 patients and the use of smart phones for 25 patients. On average the time to conduct a consultation was 14.91 ± 5.66 minutes. During the consultation the treatment of dressings was suggested to 16 patients (53.33%), while 14 patients (46.67%) were advised to undergo stepwise operative therapy. Two (6.7%) patients achieved wound healing, while others required wound dressing changes after 1 month. In Fig. 3, a 72-year-old man with a chronic wound on his left leg present for 6 years who suffered a recurrence 4 years ago. Wound debridement and split skin graft was conducted in the local hospital with telemedicine consultation. After 1 week, 95% of the grafted skin was still viable and the wound healed within 3 weeks.

**Table 1:**
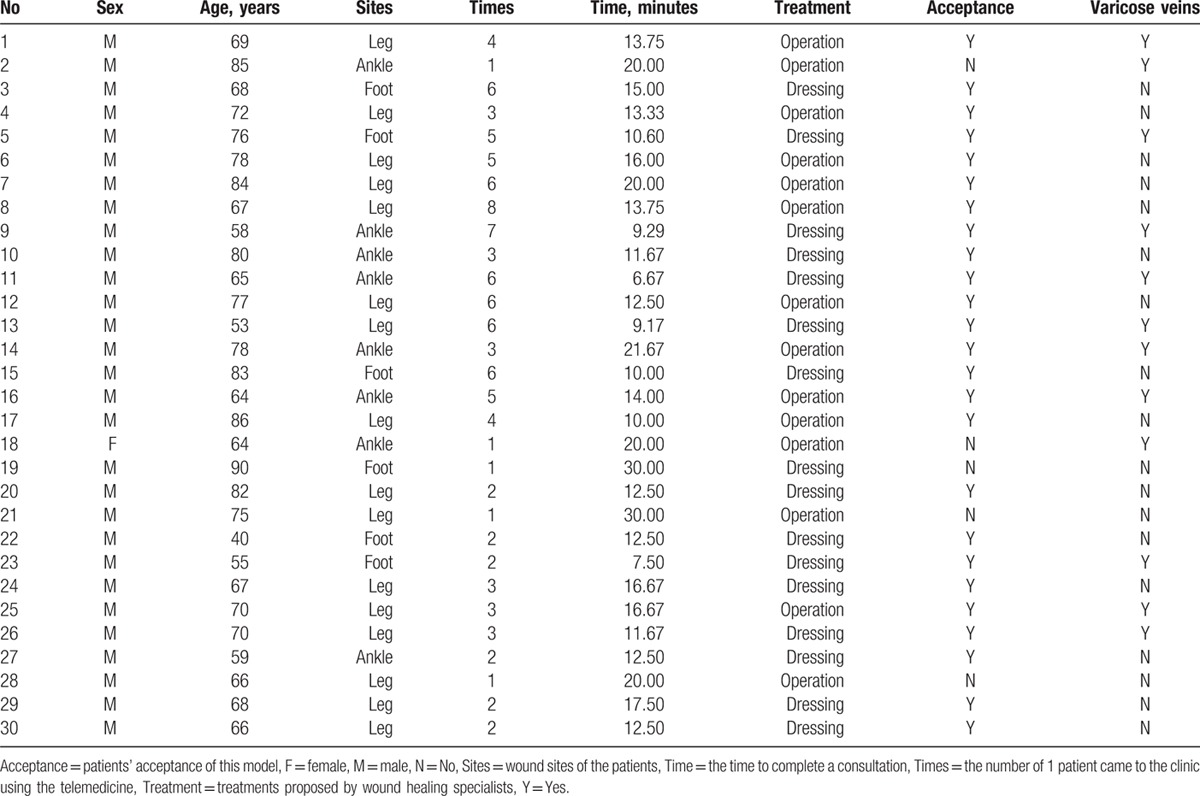
Detailed information of patients treated with this model.

Both local surgeons and patients showed good acceptance of this model (100% and 83.33%, respectively). Multiway real-time communication occurred without any difficulty. With a delay of 4 seconds or so, the upload and download speed of the Internet and Wi-Fi were satisfactory. The resolution of the video was good and both specialists and local surgeons showed good acceptance and adherence to this model. The service was free to patients as the government paid for the treatment.

## 4 Discussion

This telemedicine platform with APP of smart phones or smart glasses in a 4G context can be employed where there is 4G coverage. In the late 1950s during the advent of telemedicine, costs were high. However in developing countries, decreasing costs and increasing network coverage provide a wide range of opportunities for the APP of smart phones in daily life, which have been of particular benefit to telemedicine.^[10]^ Today's technology, specifically the mobile handsets with high portability and high-speed telecommunication systems, enables telemedicine to be inexpensive. Indeed, by reducing transportation and staff time, telemedicine decreases the costs and improves the quality of life for patients from remote and rural areas.^[25–28]^ Herein, we reported our recent experience using smart phones or smart glasses and 4G for wound management, a wound care model arose from discussions involving Shanghai and the First People's Hospital of Wucheng district, Jinhua, Zhejiang province in China. Following the introduction of this wound care model, it was deemed to be user-friendly. In general, both local surgeons and patients showed good acceptance of this model.

The benefits of this model are multidimensional. First, it provides a platform to popularize the concept of wound healing and enables local health workers to receive systematic training in wound care. In our study, local surgeons were interested and showed good compliance with the use of smart phones and smart glasses. Second, it ensures that health care can be delivered with minimal delay to those who are unable to move freely. In this study, local patients received high quality medical service near their home. Third, it is an interactive, real-time, and remote wound care strategy designed to emulate video conferencing. One case could be presented in several clinics, and doctors could discuss the case together and come to a joint decision. Effective communication between remote specialists and local surgeons is indispensable to successful telemedicine. In most cases, local surgeons follow specialists’ advice but sometimes, the local surgeons’ suggestions might be important to the therapeutic plan. Fourth, telemedicine could make fewer unnecessary referrals to specialists, which would save time and energy.

Like dermatology, a strong visual component is regarded to be valuable in wound healing as well. Most of the diagnosis and treatment of wounds are usually made by morphological observation achievable using the telemedicine system.^[29–31]^ However, the clinical history, physical examination, and auxiliary examination when necessary are still important. In one case, due to varicose veins, elastic bandages were recommended by specialists to be used. Following physical examination, 1 patient with wounds located on their foot and ankle and a diagnosis of filariasis for over 30 years was recommenced to a specialized hospital to treat lymphatic blockages first. As the study was completed over a 1-month period, most patients were still required treatment at the end of the study. These patients had longstanding wounds and were of an older population; therefore, it would take time for their wounds to heal completely.

Telemedicine has its challenges. Both smart phones and smart glasses have inherent disadvantages that limited their APP in telemedicine. As the smart phone is not hands-free, the consultation cannot be carried out when there is only 1 local surgeon at site and he is checking the wound at the same time. Similarly, due to its failing to acquire magnification of regional anatomy, local surgeons wearing smart glasses have to bring his/her face close to the wound, which has the potential to cause infection to patients. In addition, concerns on privacy and security during telemedicine have gained more and more attention in recent years.^[32,33]^ In fact, many people fear that health-related data might be misused. Such ethical and legal issues pose tremendous threat to the use of the telemedicine service. There were also reports that discussed the importance of establishing legal regulations associated with telemedicine, many of which considered that it was necessary to take effective measures to ensure security and privacy. However, it should not prevent the use of telemedicine services.^[34–36]^

In this study, a telemedicine wound care model via smart phones or smart glasses was introduced and our findings were congruent with our hypothesis. This model is particularly valuable because it provides an opportunity of high quality medical service in remote areas where wound healing specialists are scarce.

### 4.1 Limitations

Due to intention-to-treat purpose, the control group was not set up. The study was implemented in a relatively short time and the number of subjects was small. Therefore, it is difficult to come to long-term conclusions of the telemedicine system at the moment. In the future, well designed RCTs are needed to further demonstrate the effect and efficiency of telemedicine.

## 5 Conclusions

In general, this wound care model is an interactive, real-time, and remote care strategy designed to improve health care service. It is an innovative process to implement telemedicine, which can be carried out to change wound dressings or do operations with the help of expert opinions. Both local surgeons and patients showed good acceptance of this model.
